# Case of Necrosis of Lower Jaw from Mercurial Salivation

**Published:** 1871-07

**Authors:** 


### ARTICLE IX.

### *Case of Necrosis of Lower Jaw from Mercurial Salivation.*

Under the care of Dr. T. A. McGraw, Professor of Surgery in Detroit Medical College.

Thomas McGlochlin came under my care at St. Mary's Hospital on March 29th, 1870. Six years before he had been treated for syphilis, and badly salivated, had since then been well until the middle of March, when he caught a severe cold, was seized with chills, fever, headache and coryza. He consulted a physician, who ordered some powders, which he believes to have been composed of calomel. The quantity contained in each powder is unknown. After taking three or four his gums began to get very sore, his breath fœtid and his teeth loose. He, notwithstanding, continued their use according to orders, until he became alarmed.

On examination, I found him presenting every symptom of mercurial salivation in a very high degree. The peculiar odor, the ulcerated cheeks and swollen and bleeding gums, the profuse secretion of saliva, and the teeth dropping from their sockets, as well as the very positive history of the case, left no doubts regarding the diagnosis. Already, when he came under my charge, had the lower jaw-bone become seriously diseased. Sinuses discharging pus led to bone, bare of periosteum.

Chlorate of potash, in ten grain doses, was given every hour. His strength, which had begun to fail, was kept up by concentrated nourishment, given frequently in small quantities, and moderate doses of morphine served to relieve pain and produce rest. The local symptoms rapidly subsided. The swelling diminished, pain disappeared, the breath, though still fœtid, lost somewhat of its offensive character.

On April 12th, the necrosed bone seemed ready for extraction. The patient was brought before the class of the Detroit Medical College, and by forceps and fingers, more than half of the lower jaw was removed. The specimen is now in the College Museum, and represents that portion of the jaw included between the right condyle and a point half an inch to the left of the symphysis.

The patient did well, and left the Hospital free from all disease and in tolerable strength. Sufficient time had not elapsed, however, on his departure to determine whether regeneration of the bone would occur, nor have I been able to learn of his condition since, but it is remarkable that scarcely any deformity resulted from the loss of bone. The jaw seemed to retain its contour, and showed no tendency to fall in.—*Detroit Review of Medicine and Pharmacy.*

